# Influence of Sub-Inhibitory Dosage of Cefotaxime on Multidrug Resistant *Staphylococcus haemolyticus* Isolated from Sick Neonatal Care Unit

**DOI:** 10.3390/antibiotics11030360

**Published:** 2022-03-08

**Authors:** Madhurima Chakraborty, Taniya Bardhan, Manjari Basu, Bornali Bhattacharjee

**Affiliations:** 1 National Institute of Biomedical Genomics, Kalyani 741251, West Bengal, India; mc2@nibmg.ac.in (M.C.); taniya.b003@gmail.com (T.B.); 2 College of Medicine & Jawaharlal Nehru Memorial Hospital, Kalyani 741235, West Bengal, India; basu.manjari@gmail.com; 3 Amity Institute of Biotechnology, Amity University, Kolkata 700135, West Bengal, India

**Keywords:** cefotaxime, *Staphylococcus haemolyticus*, neonates, sub-MIC, biofilms, short-term evolution

## Abstract

*Staphylococcus haemolyticus* has emerged to be a frequently encountered late-onset sepsis pathogen among newborn infants. Critical care of neonates involves substantial usage of antibiotics and these pathogens are often exposed to sub-optimal doses of antibiotics which can augment maintenance of selection determinants and a range of physiological effects, prime among them being biofilm formation. Therefore, in this study, the outcome of a sub-inhibitory dosage of a commonly prescribed third-generation antibiotic, cefotaxime (CTX), on multidrug resistant (MDR) *S. haemolyticus*, was investigated. A total of 19 CTX-resistant, MDR and 5 CTX-susceptible strains isolated from neonates were included. Biofilm-forming abilities of *S. haemolyticus* isolates in the presence of sub-optimal CTX (30 μg/mL) were determined by crystal violet assays and extracellular DNA (eDNA) quantitation. CTX was found to significantly enhance biofilm production among the non-susceptible isolates (*p*-value_Wilcoxintest_—0.000008) with an increase in eDNA levels (*p*-value_Wilcoxintest_—0.000004). Further, in the absence of antibiotic selection in vitro, populations of MDR isolates, JNM56C1 and JNM60C2 remained antibiotic non-susceptible after >500 generations of growth. These findings demonstrate that sub-optimal concentration of CTX induces biofilm formation and short-term non-exposure to antibiotics does not alter non-susceptibility among *S. haemolyticus* isolates under the tested conditions.

## 1. Introduction

Coagulase-negative staphylococci (CoNS) are currently one of the major causes of device-related infections by forming biofilms, particularly in immunocompromised patients [1,2] and the methicillin-resistant species are known to be dominant colonizers among neonates admitted to intensive care units [3]. Among the CoNS species, *S. haemolyticus* is the leading cause of late-onset sepsis (LOS) in neonates and also plays a very important role in hospital-acquired infections worldwide [4,5]. Our work on nosocomial nasal colonization among preterm neonates also shows that *S. haemolyticus* is a dominant colonizer (Supplementary Table S1). *S. haemolyticus* is known for acquiring antibiotic resistance (MDR) which contributes to the establishment of more virulent clones [6] and many isolates have the ability to form biofilms [7,8,9].

Across the world, approximately 2.4 million children lose their lives in the first month of birth each year and India contributes majorly to this. India has a neonate mortality rate of 21.7% [10] and 20–41% of preterm neonates admitted to tertiary care hospitals succumb to sepsis [11], often ascribed to multidrug resistant (MDR) bacteria [12]. Among the causal pathogens, members of the *Enterobacteriaceae* and *Moraxellaceae* families dominate, but coagulase-negative staphylococci (CoNS) have been found to contribute significantly [13,14]. Along similar lines, it has been observed by the German Neonatal Network (GNN) that weekly screening for MDR colonizers has brought down the rate of LOS essentially due to the decrease in CoNS colonization [15]. It is also noteworthy to observe that with the rise in *ß-lactam* resistance and *mecA* gene carriage among clinical *S. haemolyticus* isolates, numerous combination therapies are increasingly being devised to treat LOS [16,17]. 

When bacterial populations are exposed to antibiotics, resistance clones often emerge due to chromosomal mutations or horizontal gene transfer [18]. Many groups have also shown that biofilms, in general, have considerably higher tolerance to antimicrobial agents as compared to planktonic cells [19,20]. Exposure to sub-optimal concentrations of antibiotics does not inhibit bacterial growth [21]. However, given that each class of antibiotic targets a molecule or enzyme with an essential function, it has been observed that sub-MIC doses of antibiotics influence bacterial pathogenicity, stress response, motility, and biofilm formation [22]. Along similar lines, it has been shown that sub-lethal concentrations of an alcohol-based disinfectant also enhances multi-species biofilms when pathogens are exposed to different stress factors i.e., antimicrobial exposure and nutrient-poor environments [23]. Twenty-seven percent of erythromycin-resistant *Staphylococcus epidermidis* have been found to exhibit biofilm induction by 0.25 MIC [24]. Kaplan et al. showed biofilm induction in the presence of low dose of *β-lactam* antibiotics in *Staphylococcus aureus* strains [25,26]. In yet another study, biofilm formation was found to be enhanced by sub-inhibitory concentrations of cell wall synthesis inhibitors in *Enterococcus faecalis* [27]. Several studies have also found that sub-optimal doses of antibiotics can induce biofilm development in Gram-negative species [28,29].

The Sick Newborn Care Units (SNCUs) are known for considerable use of antibiotics and empiric use is also rampant [30]. Further, it is also unclear as to how many generations it would take after discontinuation of an antibiotic for a resistant bacterial population to become susceptible again [31]. To address each of these questions, we focused on a known LOS pathogen, *S. haemolyticus*. The experiments were carried out to understand if a sub-optimal dosage of one of the most prescribed antibiotics, cefotaxime (CTX), a third-generation cephalosporin, results in enhanced biofilm formation in the laboratory. Finally, short-term 15-day evolution experiments were carried out to evaluate if discontinuation of the antibiotic could result in loss of non-susceptibility.

## 2. Results

### 2.1. Susceptibility Profiling

**Figure 1:**
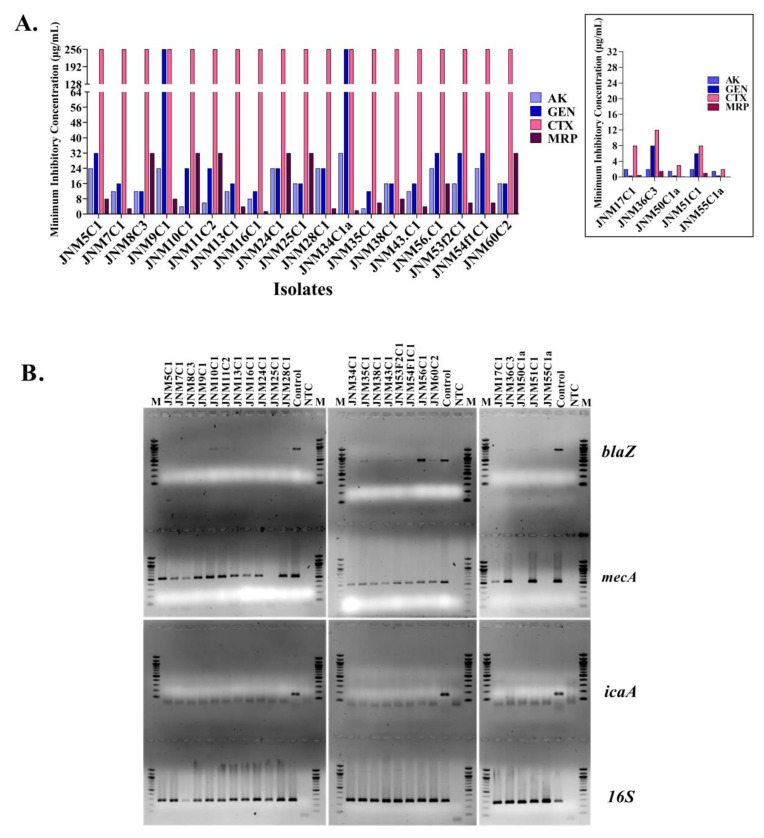
Antimicrobial susceptibility and gene profiles of 19 CTX non-susceptible *S. haemolyticus* isolates. (**A**). The minimum inhibitory concentration of (MIC) of the 19 non-susceptible *S. haemolyticus* isolates with the inset showing the MIC of the five susceptible isolates. (**B**). The *blaZ* (858 bp), *mecA* (533 bp) and N-acetylglucosaminyltranferase (*icaA*) (166 bp) amplicons were run on 1.5% agarose gels. M; 100 bp marker, control; positive control, NTC; no template control. *16S* amplicon (292 bp) served as control.

The antibiotic susceptibilities of a total of 24 *S. haemolyticus* isolates were characterized to identify the MDR strains and appropriate controls. A total of five antibiotics including CTX were tested. Nineteen isolates (79.16%) were non-susceptible to CTX with MIC values of >256 µg/mL followed by MRP (*n* = 13, 54.17%; MIC: 6–32 µg/mL). A total of 18 out of 19 isolates harbored the *mecA* gene (94.74%) and 11 were amplification positive for the *blaZ* gene (57.89%). Out of the five CTX sensitive isolates, all were susceptible to AK, MRP and three were susceptible to GEN (Figure 1A, Supplementary Table S2). Two CTX susceptible isolates with MIC of 8 µg/mL (JNM17C1 and JNM51C1) were also found to harbor the resistance genes (Figure 1B, Supplementary Table S2). 

### 2.2. Biofilm Enhancement and eDNA Release among the Isolates

**Figure 2:**
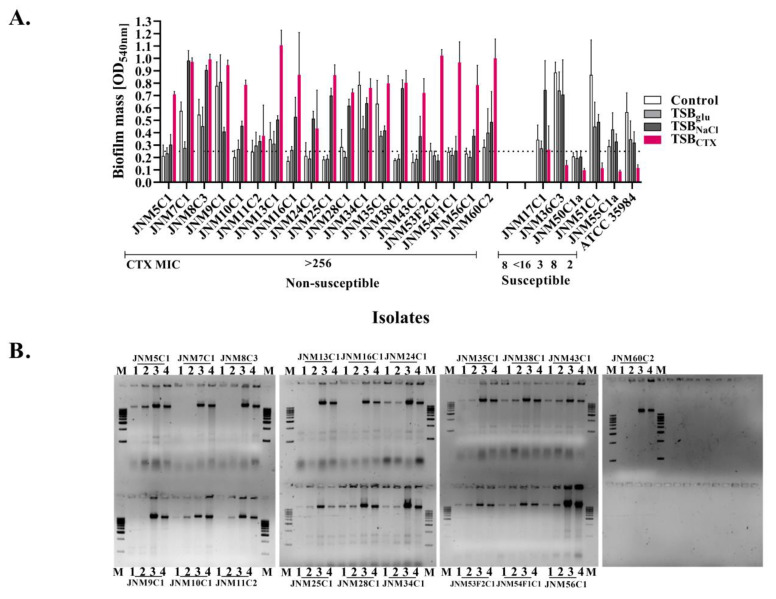
Cefotaxime (CTX), a commonly used *ß-lactam* antibiotic enhances biofilm formation among non-susceptible *S. haemolyticus* isolates in vitro. (**A**). Semi-quantitative determination of 24 h biofilm formation under known inducible conditions (tryptic soy broth with 1% glucose (TSB_glu_) and 3% sodium chloride (TSB_NaCl_)) and 30µg/mL CTX(TSB_CTX_) using crystal violet assays. The dotted line denotes the cut-off optical density value for biofilm formation (0.25). The biofilm-forming ATCC 35984 *S. epidermidis* strain served as a control. (**B**). Biofilm supernatants run on 0.8% agarose gels to determine the presence of extracellular DNA (eDNA). M; 1000 bp marker. The lanes labelled 1, 2, 3 and 4 in each case denote TSB, TSB_glu_, TSB_NaCl_ and TSB_CTX_, respectively.

In the presence of sub-inhibitory concentrations of CTX (30μg/mL), biofilm production was enhanced significantly (*p*-value_Wilcoxintest_—0.000008) among all the non-susceptible isolates. By contrast, there was a reduction in median optical density values among the susceptible isolates, presumably due to cell death (Figure 2A, Supplementary Table S3). All except for one isolate (JNM50C1a) grown in TSB_glu_ and TSB_NaCl_ were identified to be biofilm producers after 24 h. The growth media strongly influenced biofilm formation and a total of 14 out of 24 (58.33%) isolates produced biofilms in TSB_glu_ whereas 22 (91.67%) isolates formed biofilms in TSB_NaCl_.

Given that eDNA release has been implicated in *S. haemolyticus* biofilm formation, whether increased biofilm formation also resulted in an increase in quantities of eDNA in the biofilms was next evaluated. Biofilm-forming sessile cells were harvested and removed by centrifugation and the cell-free nucleic acid in the supernatant was harvested and resolved on 0.8% agarose gels (Figure 2B). Significant increases in eDNA levels were observed in the TSB_NaCl_ (*p*-value_Wilcoxintest_—0.000004) and TSB_CTX_ (*p*-value_Wilcoxintest_—0.000004) treated groups (Supplementary Table S4). 

### 2.3. Whole Genome Sequencing and Resistome Mapping of Ancestral Populations

To generate the resistome profiles of two MDR *S. haemolyticus* ancestral populations (JNM56C1 and JNM60C2), both non-susceptible to CTX, GEN and MRP, paired-end whole genome sequencing was carried out. Iterative de novo and reference guided assembly (NC_007168) resulted in alignment of >93% of error-corrected reads. JNM56C1 was determined to be a multi locus sequence type (ST) 38 with a chromosome length of 2,554,979 bp (CP063753) at an average sequencing depth of 310. A total of six antimicrobial resistance genes were annotated, namely *AAC(6′)-Ie-APH(2″)-Ia*, *blaZ*, *dfrG*, *mecA*, *msrA* and *mphC*. Similarly, JNM60C2, a ST-1 isolate, had a chromosome sequence length of 2,511,057 (CP065356) which was ascertained at an average depth of 421. The antimicrobial genes identified were *APH(3′)-IIIa*, *AAC(6′)-Ie-APH(2″)-Ia*, *blaZ*, *dfrG*, *mecA* and *sat-4*. The polysaccharide intercellular adhesin (PIA) operon was confirmed to be absent from both the genomes. None of the isolates were found to harbor any plasmids. 

### 2.4. No Change in Susceptibility in the Absence of Antibiotic Selection

**Figure 3:**
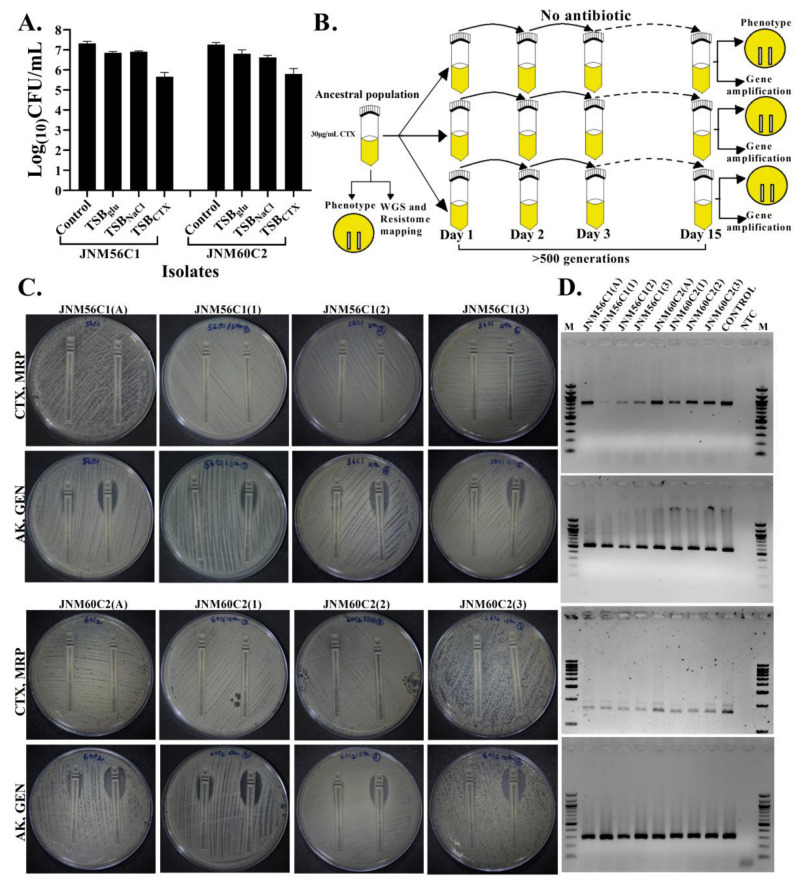
No change in in vitro antibiotic susceptibility among MDR, biofilm-forming *S. haemolyticus* isolates (JNM56C1 and JNM60C2) grown in the absence of antibiotics in short-term evolution experiments. (**A**). The presence of living sessile cells is confirmed by log(10)CFU counts in both isolates in the presence of 30 μg/mL CTX. The error bars represent standard deviation across three technical replicates. (**B**). Study design of the evolution experiment. (**C**). Comparison of susceptibility to *ß-lactam* and aminoglycoside antibiotics among ancestral and evolved populations using E-tests. (**D**). The *blaZ* (858 bp, panel 1), *mecA* (533 bp, panel 2) and *AAC(6′)-APH(2′)*(1658 bp, panel 3) and *16S* (292 bp, panel 4) genes were amplified from the ancestral and evolved populations and amplicons were run on 0.8–1.5% agarose gels. M; 100/1000 bp marker, control; positive control, NTC; no template control.

It has been shown in *S. aureus* strains using in vitro curing assays that harboring SCCmec imposes a fitness cost [32]. However, if a similar cost is attached with non-susceptibility in *S. haemolyticus*, a decay of non-susceptibility in the absence of antibiotic selection needs to be tested. To understand this, JNM56C1 and JNM60C2, two completely sequenced multidrug resistant *S.haemolyticus* populations with biofilm-forming ability (Figure 3A) and known generation time of 30 and 40 min, respectively (Supplementary Table S5), were serially passaged in triplicate for >500 generations in the absence of antibiotics (Figure 3B). Susceptibility to CTX was then compared among the six evolved clones and the two ancestral populations along with candidate resistance gene amplification. Additionally, meropenem (MRP), amikacin (AK) and gentamicin (GEN) susceptibilities were also compared. There were no differences in CTX susceptibility. Similarly, MRP, AK and GEN susceptibilities remained unchanged (Figure 3C). In all evolved populations, resistance genes *blaZ*, *mecA* and *AAC(6′)-APH(2′)* were retained (Figure 3D).

## 3. Discussion

*S. haemolyticus* is an emerging nosocomial pathogen known for multidrug resistance and severe outcomes among preterm neonates and immunocompromised individuals [6,33]. Blood stream and device-linked infections are commonly reported and the virulence determinants include biofilm formation along with production of modulins and rearrangements attributed to insertion sequences [34]. CTX, on the other hand, is a third-generation cephalosporin included among the first line of WHO recommended antibiotics in neonatal sepsis [35]. This study was undertaken to understand if an in vitro equivalent of the therapeutic dose of CTX (30 µg/mL) [36] could affect the physiology of resistant *S. haemolyticus* colonizers. Biofilm formation and presence of antibiotic-resistance genes (ARGs) *mecA* and *blaZ* reiterated the invasive phenotype of the clinical isolates included in this study [34]. We chose to delineate biofilm formation as the physiological effect as it is known to accentuate antibiotic resistance and colonization. 

The sessile aggregates of biofilm-associated cells within the meshwork of extra-cellular polymeric matrix are known for enhanced resilience to antibiotics due to decreased penetration and presence of metabolically inert persister cells [37]. Thus, escalation of biofilm formation by any external agent can essentially skew a bacterial disease towards poor prognosis. Sub-inhibitory concentration antibiotics belonging to multiple classes have been reported among a number of diderms such as *Acinetobacter baumannii*, sensitive *Campylobacter jejuni*, *Leptospira* spp. and *Pseudomonas aeruginosa* [28,38,39,40].Changes in morphology with exposure to sub-optimal concentrations of penicillins have been reported as early as 1975 in the presence of intact cell division [41]. Among the monoderms, enhancement has also been reported upon the use of cell wall synthesis inhibitors including *ß-lactam* antibiotics among *E. faecalis* and *S. aureus* isolates with release of extracellular DNA (eDNA) [26,27]. However, reports on *S. haemolyticus* are lacking, though this coagulase-negative *Staphylococcus* species lacking the PIA operon has been shown to form biofilms in vitro under different growth conditions [42,43]. There exists only a single report where nosocomial clones were found to show enhanced biofilm formation on glass and polystyrene surfaces in the presence of one-fourth MIC of three antibiotics, namely oxacillin, vancomycin, and linezolid [7].Therefore, our study is one of the first showing increase in biofilm formation and eDNA release among MDR, nosocomial *S. haemolyticus* isolates in the presence of sub-inhibitory concentrations of CTX. 

Evolved antibiotic resistance is a costly affair for many species of bacteria and often results in decreased competitive fitness in the absence of selection pressure [44,45]. Nevertheless, it has also been exhibited that *S. aureus* often tackles such trade-offs by way of mutations that compensate for changes [46]. Furthermore, in long-term evolution, experiments with *E.* *coli* populations have also shown that there is no change in susceptibility [47]. Along similar lines, our short-term evolution experiments (>500 generations) in the absence of selection pressure resulted in no change in MIC values of CTX and MRP in the evolved clones. However, we believe that a better approach would have been whole genome sequencing of the evolved clones instead of a candidate gene approach to gauge the entire repertoire of genetic changes incurred by the evolved populations.

## 4. Materials and Methods

### 4.1. Minimum Inhibitory Concentration (MIC) Determination of S. haemolyticus Isolates

The *S. haemolyticus* strains included in this study were isolated from nasal swab samples collected from neonates admitted after birth to the Sick Neonatal Care Unit (SNCU) of College of Medicine & JNM Hospital, Kalyani between 2017–2018 as approved by the Hospital and Institutional Ethical Committees. Consenting mothers signed a consent form prior to sample collection. The minimum inhibitory concentration (MIC) values were determined using MIC strips (Himedia Labs). The antibiotics tested were CTX, aminoglycosides, AK, GEN, carbapenem and MRP. The Kirby–Bauer disc diffusion assay was generated for the antibiotics fluoroquinolones; ciprofloxacin (CIP). Experiments were carried out according to Clinical and Laboratory Standards Institute (CLSI) guidelines [48].

### 4.2. Quantification of Biofilms

The biofilm-forming ability of *S. haemolyticus* isolates was determined by a modified crystal violet assay method as described previously [43]. Briefly, 96-well polystyrene, flat-bottom microtiter plates were filled with 180 μL of tryptic soy broth (TSB) (Himedia labs) and 20 μL bacterial cells grown to a Macfarland score of 0.5 in brain heart infusion broth (BHI) (Himedia labs) were added and incubated at 37 °C for 24 h statically. Biofilm-forming capacities of all isolates were determined in TSB, TSB with 1% glucose (TSB_glu_), TSB with 3% NaCl (TSB_NaCl_), and TSB with 30 μg/mL CTX (TSB_CTX_) which is an in vitro equivalent of the therapeutic dosage [36]. After 24 h, planktonic cells were removed, adherent cells were fixed with 99% methanol (Finar chemicals, Ahmedabad, India) for 10 min, and plates were washed once with 1× PBS (Sigma-Aldrich, St. Louis, MO, USA) and air-dried for 10 min. Modified crystal violet assays were performed, and the absorbance (OD) was recorded at 540 nm. The assays were performed with 6 replicates for each condition in 2 parallel runs. The isolates with an OD of ≥0.25 were considered biofilm positive. *S. epidermidis* ATCC 35984 was used as a positive control. The presence of living sessile cells was determined by colony-forming units (CFU) for selected isolates in the presence of TSB_glu_, TSB_NaCl_ and TSB_CTX_ in triplicate.

### 4.3. Extracellular DNA (eDNA) Quantification 

Extracellular DNA was extracted as previously described by Kaplan et al. with modification [25]. Biofilms were grown in triplicate in TSB, TSB_glu_, TSB_NaCl_ and TSB_CTX_ in 24-well polystyrene microtiter plates, in a total volume of 1 mL per well. After 24 h of growth, the liquid was carefully removed, the plates were washed once with 1XPBS (Sigma-Aldrich, St. Louis, MO, USA) and 50 μL of Tris-EDTA buffer (10 mM Tris-HCl, 1 mM EDTA, pH 7.4) (Sigma-Aldrich, St. Louis, MO, USA) was added to each well. The biofilm-forming cells were scraped off the bottom surface of the wells and were transferred to 1.5 mL microcentrifuge tubes. The tubes were centrifuged at 13,000 rpm for 25 s and 8 μL of each of the supernatants were resolved on 0.8% agarose gels. Densitometric analyses of eDNA were carried out using Image Lab software version 6.0.1(Bio-Rad Laboratories, Hercules, CA, USA).

### 4.4. Genomic DNA Isolation and Whole-Genome Sequencing

Two isolates (JNM56C1 and JNM60C2) were subcultured in the presence of 30 μg/mL CTX. Total DNA from both were purified using the QIAamp DNA extraction mini kit (Qiagen, Hilden, Germany) and were subjected to paired end whole genome sequencing (2 × 300 bp) on an Illumina HiSeq2500 platform (Illumina, San Diego, CA, USA). Both de novo and reference guided assembly was carried out using Velvet and Bowtie2, respectively [49,50], to build genomes as described previously [51].

### 4.5. Short-Term Evolution Experiment

The isolates grown in the presence of 30 μg/mL CTX formed the ancestral population for each. Generation times for both were calculated from growth curves. The two populations were serially passaged for >500 generations in triplicate for 15 days in fresh Luria–Bertani (LB) media (Himedia labs) in the absence of antibiotic selection at a dilution of 1:100. The ancestral and evolved populations were tested for antibiotic susceptibility for *β-lactam* (CTX, MRP) and aminoglycoside (AK, GEN) antibiotics by Etests.

### 4.6. Amplification of Genes

The presence of *β-lactamase* genes *mecA* and *blaZ*, aminoglycoside resistance gene *AAC(6′)-APH(2′)*, and N-acetylglucosaminyltransferase *icaA* gene were detected by using PCR amplification. The positive controls used were a laboratory isolate for the *β-lactamase* and aminoglycoside resistance gene and *S. epidermidis* ATCC 35984 for *icaA* gene amplification. A 292 basepair (bp) region of the *16S* gene was amplified as an internal control. Supplementary Table S6 lists all the primers used in this study. 

### 4.7. Statistical Analyses

Normality across datasets was evaluated using the Kolmogorov–Smirnov test. To identify significant differences among experimental conditions tested, the Wilcoxon test was performed using GraphPad Prism version 9.1.2 (GraphPad Software, La Jolla, CA, USA). A *p*-value of <0.05 was considered to be statistically significant. 

## 5. Conclusions

MDR *S. haemolyticus* is a late-onset sepsis pathogen which has observed to be a predominant nasal colonizer among hospitalized preterm neonates. This study demonstrates that these CTX non-susceptible, MDR, biofilm-forming *S. haemolyticus* isolates exhibit enhanced biofilm formation upon exposure to a therapeutic dosage of a commonly used antibiotic, CTX, in vitro. Increase in eDNA release implicated in biofilm formation was significantly higher upon sub-MIC CTX usage. Further, no changes in susceptibility to both commonly used *β-lactams* and aminoglycosides were observed among non-susceptible isolates when grown in the absence of selection pressure in the short-term. All these findings together reiterate the need for antibiotic stewardship. 

## Data Availability

The annotated genome sequences (CP063753, CP065356) and the *16S* amplicon sequences (MZ636452-MZ636490) are available at the GenBank database.

## Supplementary Materials

The following supporting information can be downloaded at: https://www.mdpi.com/article/10.3390/antibiotics11030360/s1, Table S1: Summary of preterm neonate subjects and species identification **; Table S2**: Summary of MIC values and resistance gene detection; Table S3: Median optical density values obtained from crystal violet assays run to determine biofilm formation; Table S4: eDNA quantitation values of 24-h biofilms; Table S5: Summary of generation time values of isolates JNM56C1 and JNM60C2; Table S6: Information on the primer sets used in this study.
